# Combined leaching and plant uptake simulations of PFOA and PFOS under field conditions

**DOI:** 10.1007/s11356-020-10594-6

**Published:** 2020-08-31

**Authors:** Matthias Gassmann, Eva Weidemann, Thorsten Stahl

**Affiliations:** 1 Department Hydrology and Substance Balance, University of Kassel, Kassel, Germany; 2 Chemical and Veterinary Analytical Institute Münsterland-Emscher-Lippe, Münster, Germany

**Keywords:** PFASs, PFAAs, Perfluoroalkyl substances, MACRO model, Non-extractable residues, Irreversible adsorption, Field lysimeter, PFOA, PFOS

## Abstract

Per- and polyfluoroalkyl substances (PFASs) are used in industrial production and manufacturing but were repeatedly detected in agricultural soils and therefore in cash crops in recent years. Dissipation of perfluoroalkyl acids (PFAAs), a sub-group of PFASs, in the environment was rather attributed to the formation of non-extractable residues (NER) than to degradation or transformation. Currently, there are no models describing the fate of PFAAs in the soil-plant continuum under field conditions, which hampers an assessment of potential groundwater and food contamination. Therefore, we tested the ability of the pesticide-leaching model MACRO to simulate the leaching and plant uptake of perfluorooctanoic acid (PFOA) and perfluorooctane sulfonate (PFOS) in a field lysimeter using two concepts of adsorption: a kinetic two-side sorption concept usually applied for pesticide leaching (scenario I) and the formation of NER (scenario II). The breakthrough of substances could be simulated adequately in scenario II only. Scenario I, however, was not able to reproduce sampled leaching concentrations. Plant uptake was simulated well in the first year after contamination but lacked adequacy in the following years. The model results suggest that more than 90% of PFOA and PFOS are in the pool of NER after 8 years, which is more compared with other studies. However, since NER formation was hypothesized to be a kinetic process and our study used a PFASs leaching time series over a period of 8 years, the results are reasonable. Further research is required on the formation of NER and the uptake of PFAAs into plants in order to gain a better model performance and extend the simulation approach to other PFAAs.

**Electronic supplementary material:**

The online version of this article (10.1007/s11356-020-10594-6) contains supplementary material, which is available to authorized users.

## Background

Per- and polyfluoroalkyl substances (PFASs) are used in industrial production and manufacturing for various purposes, mainly due to their surfactant, water and oil repellent, and catalytic and stable nature (3M 2000). In recent years, widespread contamination of soils, groundwater and eventually drinking water wells was identified at various places in Europe (Banzhaf et al. 2017; Biegel-Engler et al. 2017; Zafeiraki et al. 2015). Sources of PFAS in soils include manufacturing sites (Hansen et al. 2002), atmospheric deposition (Taniyasu et al. 2013), landfills (Lang et al. 2017), sewage sludge (Stahl et al. 2018) and fire extinguishing foam (Moody and Field 2000). In Germany, two cases have been identified in which agricultural fields and underlying groundwater were contaminated by repeated application of organic fertilizer containing PFAS (Biegel-Engler et al. 2017; Stahl et al. 2012). Among all chemicals belonging to the group of PFAS, most are not yet investigated in environmental studies. However, two perfluoroalkyl acids (PFAAs), a sub-group of PFASs (Buck et al. 2011), have repeatedly been measured in the environment and investigated in the lab: perfluorooctanoic acid (PFOA) belonging to the group of PFCA (perfluoroalkyl carboxylic acids) and perfluorooctane sulfonate (PFOS) belonging to the group of PFSA (perfluoroalkyl sulfonic acids) (Buck et al. 2011). Thus, these substances are generally treated as ‘lead substances’ for PFAAs in environmental studies.

In contrast to other organic chemicals, most PFAAs are resistant to hydrolysis, photolysis and microbial degradation in the environment (3 M 2000; UNEP 2006; Prevedouros et al. 2006; Du et al. 2014; ECHA 2018). Sorption strength (i.e. the distribution coefficient *K*_*d*_) of PFAAs is positively correlated with the length of their molecular carbon chain (Gellrich and Knepper 2012). In several studies, *K*_*d*_ was linearly correlated to the fraction of organic carbon (OC) in soils indicating that OC is the major sorbent material (Milinovic et al. 2016; Miao et al. 2017). However, these linear correlations might have positive intercepts which can be interpreted as background sorption due to other sorbents such as mineral soil components (Milinovic et al. 2016). Sorption isotherms fitted to experimental PFAAs sorption data are usually of Freundlich type (Milinovic et al. 2016), Langmuir type (Hellsing et al. 2016) or both (Hansen et al. 2002; Ochoa-Herrera and Sierra-Alvarez 2008), whereas some studies found a linear isotherm (Milinovic et al. 2016; Ahrens et al. 2011) and others non-linear isotherms (Hansen et al. 2002) or both (Ochoa-Herrera and Sierra-Alvarez 2008). At experimental substance concentrations in the ng/l and μg/l range, all isotherm types indicate that the maximum loading of sorption sites in the considered soil was not yet reached. At higher concentrations (> 1 mg/l), the exponent of the Freundlich isotherm decreased indicating stronger competition for sorption sites (You et al. 2010). The lower concentration ranges represent environmental concentrations, and the higher concentrations are used to determine the maximum loading on the sorption sites (Hansen et al. 2002). The dissipation of PFAAs in soils, even though they are not degraded, was repeatedly explained by the formation of non-extractable residues (NER) (McLachlan et al. 2019). The degree of NER formation was found to be dependent on the *K*_OC_ (organic carbon normalized sorption coefficient), resulting in a higher fraction of NER of, e.g. PFOS (40–98% after 24 h) compared with PFOA (0–75% after 24 h) in short-time (days) equilibrium batch experiments (Chen et al. 2016; Milinovic et al. 2016). However, the opposite was reported from a field lysimeter study: after a 4-month leaching experiment, about 20% of PFOS could not be recovered, while PFOA dissipated to about 45% (McLachlan et al. 2019). The formation of NER was further suggested to be a time-depended process (McLachlan et al. 2019). In contrast, a recovery of short-chain PFAAs (up to 6 (PFCA) and up to 5 (PFSA) fluorinated C atoms (Buck et al. 2011)) of 80 to 90% was found in column experiments in a time span of 2 years, indicating largely reversible adsorption (Gellrich et al. 2012). Therefore, even though PFAAs are not degraded in the soil environment like pesticides, they partially dissipate by building NER. Still, similar to pesticides, PFAAs are at least partially reversibly adsorbed to soil particles, which can be described by sorption isotherms.

Besides groundwater and thus potential drinking water contamination, substance uptake from soil solution to plant roots is a major entry of PFAAs into the food chain. Generally, the uptake amount differs between different plant species (Stahl et al. 2009). In lettuce, the concentration of PFOA and PFOS was more than 10 times lower in the transpiration stream of the plants compared with the pore water in soil (Felizeter et al. 2012).

Up-to-date leaching models for organic chemicals include a set of known environmental fate process descriptions for transport, transfer (e.g. sorption, uptake) and transformation of substances. In contrast to PFAAs assessment, leaching simulations are part of exposure assessment of the fate and effect of pesticides and veterinary pharmaceuticals in groundwater in the regulatory assessments of the European Union (European Commission (EC) 2009, 2019). One of the models used for this purpose is the MACRO model (Larsbo et al. 2005). The model was applied for leaching in different soils, for different pesticides and different conservative substances (inert hydrological tracers) (Larsbo and Jarvis 2005). One attempt for simulating other organic chemicals with MACRO was an application for veterinary pharmaceuticals (Larsbo et al. 2008). PFAAs leaching potential was simulated in a field lysimeter using the PELMO model (McLachlan et al. 2019). For this purpose, the dissipation of substances was not simulated, but the leaching was only determined by soil properties and sorption coefficients. Another leaching simulation of PFOA was performed using the PRZM model in a modelling chain, also neglecting NER formation (Shin et al. 2012). The assessment of model fit was hampered by highly uncertain PFOA input values. In unsaturated laboratory soil columns, several reversible mass transfer processes such as adsorption at the air-water interface were used for a simulation of PFAAs leaching (Brusseau 2020). Thus, even though NER formation was repeatedly found in experimental studies, this process was not included in the simulation of PFAAs leaching up until now. Furthermore, existing PFAAs modelling studies were restricted to short-term experiments. Finally, there is no modelling approach in the literature for the simulation of PFAAs leaching and plant uptake under field conditions with sufficient evidence of applicability.

Therefore, this study investigated the applicability of a pesticide-leaching model to simulate PFAAs fate and transfer in soil and into plants considering NER formation. For that purpose, data of a long-term lysimeter study regarding PFOA and PFOS leaching (Stahl et al. 2013) and an update of sampling data up to the year 2015 is used for the calibration of the leaching model MACRO 5.2 for both PFAAs.

## Methods

### Prior lysimeter experiment

The behaviour of PFAAs was studied in a lysimeter experiment between 2007 and 2015 in Kassel, Germany. Technical mixtures of 360 g PFOA (96% purity) and 367.5 g PFOS (98% purity) were applied once at the end of March 2007 to four identical 1 m^2^ square-sized and 1.5-m deep lysimeters containing undisturbed soil columns (monolithic soil columns). This application mass represents rather a worst-case spill (25 mg/kg soil) than a background pollution, which was found to be in the μg/kg range for the sum of PFAS in German soils (Kotthoff et al. 2020). The leachate, which was caused by natural rainfall, was collected monthly with glass drain bottles (each with a capacity of 60 L) and analysed for PFOA and PFOS in the lab. The crop rotation at the lysimeter varied every year (winter wheat (2007), winter rye (2008), canola (2009), winter wheat (2010), winter barley (2011), canola (2012), winter wheat (2013), winter barley (2014) and winter rye (2015)). Crops were grown under consideration of agricultural practices such as fertilization. After harvesting straw and grain were separately analysed. The lysimeter experiment is described in detail elsewhere (Stahl et al. 2013). The soil parameters of the medium to highly silty soil can be taken from the supplement Table 6.

### The macro model

The MACRO model is a one-dimensional numerical leaching model being able to simulate water fluxes and reactive solute transport in soils (Larsbo and Jarvis 2003). MACRO 5.2 can be downloaded as a command-line version from the developer’s website (SLU 2020), which facilitates its use in optimization algorithms. Simulation results can be analysed using the Macroutils R package (Moeys 2015). Water fluxes are calculated in MACRO using a dual porosity domain, including micropore and preferential macropore flow. Evapotranspiration is determined by using the Penman-Monteith approach. For this study, especially, the reactive transport module was tested, and thus, only corresponding relevant equations are elaborated below. For further reading, the technical description of MACRO 5.2 is recommended (Larsbo and Jarvis 2003).

Solute transport in the soil matrix is determined by the advection-dispersion equation. The dispersion coefficient *D* is calculated using the diffusion coefficient in free water and the dispersity of the soil *D*_*v*_. Sorption equilibrium is calculated by a Freundlich type isotherm:

1 $$ s={K}_f\cdotp {c}_{\mathrm{soil}}^n $$

where *s* is the concentration in the adsorbed phase, *c*_*soil*_ is the concentration in the dissolved phase, *K*_*f*_ is the Freundlich sorption coefficient and *n* is the Freundlich exponent. *K*_*f*_ can be calculated from *K*_fOC_, the organic-carbon normalized sorption coefficient, and the organic carbon content *f*_OC_ of soil by

2 $$ {K}_f={K}_{\mathrm{fOC}}\cdotp {f}_{\mathrm{OC}} $$

Sorption kinetics is either calculated in the model by an instantaneous equilibrium or by first-order sorption kinetics, considering an equilibrium and a non-equilibrium fraction *f*_ne_ of sorption sites. The kinetic rate *α*_*k*_ of adsorption and desorption is the same. The concentrations in the non-equilibrium phase *c*_ne_ are calculated from:

3 $$ \frac{\partial {c}_{\mathrm{ne}}}{\partial t}={\alpha}_k\cdotp \left(\rho \cdotp {c}_{\mathrm{soil}}-\frac{c_{\mathrm{ne}}}{f_{\mathrm{ne}}}\right) $$

where *ρ* ist the bulk density of the soil. The degradation of substances is described by a first-order equation with degradation rate *r*:

4 $$ \frac{\partial c}{\partial t}=r\cdotp c $$

where *r* can be calculated from a half-life DT_50_ by $r=\frac{\ln (2)}{{\mathrm{DT}}_{50}}$. The degradation rate is influenced by soil moisture and soil temperature considering the effects of both variables on microbial activities. Degradation can be calculated in four compartments: the adsorbed phase in micro- and macropores and the dissolved phase in micro- and macropores. Both, adsorption and degradation parameters, can be specified for each soil layer. In this study, we kept the parameters constant with depth, except *K*_*f*_ which was calculated by *f*_OC_ of each layer.

Root uptake *U* of chemicals is calculated as mass transfer due to water uptake by transpirative forces and a factor, the transpiration stream coefficient (*f*_star_), determining the fraction of concentrations in the roots *c*_root_ compared with the soil water *c*_soil_:

5 $$ {f}_{\mathrm{star}}=\frac{c_{\mathrm{root}}}{c_{\mathrm{soil}}} $$

Considering the implemented equations, the model availability and the additional analytical tools, MACRO is a state-of-the-art leaching model which is relatively easy to apply.

### Model setup and input data

The MACRO model was set up with the boundary condition of a free-draining lysimeter and an annual crop rotation. The option of a solute fate model for pesticides was used in MACRO, and the lysimeter was assumed free of PFAAs at the beginning of the simulation. Both substances were applied in the model at the 31th of March 2007 with the same masses as in the lysimeter study. One year of model warm-up was simulated before substance application to gain a realistic soil water distribution in the lysimeter. The leaching was determined until May 2015.

The soil parameters required for the simulation were calculated by means of the MACRO pedotransfer functions included in the software using basic physical soil properties. All parameters can be found in the supplement Table 4 to Table 6. Plant growth parameters were set according to different sources including the FOCUS (FOrum for Co-ordination of pesticide fate models and their USe) Scenario D4, which is used in the regulatory assessments of pesticides in the European Union (Table 4 in the supplement). Daily meteorological data was taken from the German weather service for the station Kassel (2006–2013) and the station Schauenburg-Elgershausen (2013–2015), which was installed as the successor by the weather service (DWD 2007–2015).

**Fig. 1:**
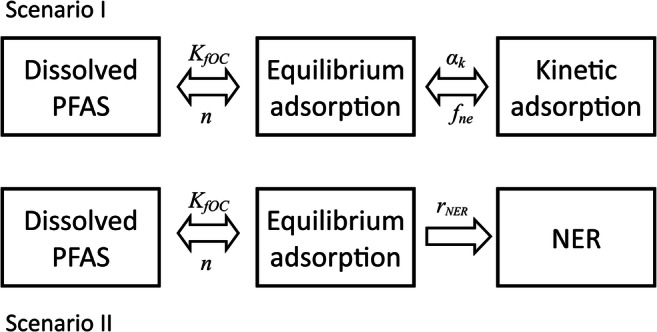
Sorption concepts implemented in the two scenarios and their corresponding parameters (NER, non-extractable residues)

In contrast to pesticides, PFAAs are not degradable in the environment. Thus, degradation is not considered in the model setup of this study. Since the discussion about NER formation and the nature of its irreversibility are ongoing and controversial, we set up the model with two different sorption concepts (Fig. 1):

Scenario I. Adsorption includes a spontaneous reversible and a kinetic reversible fraction.
Scenario II. Adsorption includes a spontaneous reversible and a non-reversible fraction (NER). The non-reversible adsorption is time-dependent.

Since MACRO does not allow for the calculation of NER formation, we set up a work-around solution: first, an instantaneous equilibrium reversible adsorption was calculated, and then the substance located in the adsorbed phase was degraded, mimicking a kinetic fixation. For this purpose, the dependency of the degradation equation on soil moisture and soil temperature was switched off in MACRO. PFAAs dissipation was thus described by the kinetic rate parameter *r*_NER_.

### Modelling strategy

A total of four substance parameters, two plant parameters and two site parameters were chosen for calibration of the model. The ranges of PFAAs environmental fate parameters are taken from the literature as specified in Table 1. The kinetic rate of NER formation could not be taken from the literature but was initially estimated between 0.001 and 0.014 1/d which equals a half-life time of 1000–50 days. The kinetic rate of adsorption was optimized in a wide range of 0.0001–0.1 1/d in order to cover realistic kinetic rates slower than instantaneous adsorption. The transpiration stream coefficients are usually calculated from *K*_OW_ (Briggs et al. 1982). However, since *K*_OW_ cannot be calculated for PFAAs, we had to make an educated guess from preliminary manual simulations. Finally, the site albedo as a parameter influencing evapotranspiration in MACRO was estimated to be in a range of 0.05–0.25.

**Table 1:** Parameters and values considered in calibration

Parameter		Unit	PFOA	PFOS
*r*_NER_	Kinetic rate of NER formation	1/d	0.001–0.014^a^	
*α*_*k*_	Kinetic rate of adsorption	1/d	0.0001–0.1^a^	
*f*_ne_	Fraction of kinetic sorption sites	-	0–1	
*K*_fOC_	Sorption coefficient	ml/g	22–332^b^	254–1176^b^
Freundlich *n*	Freundlich exponent	-	0.78–1.27^c^	0.6^d^–1.0^e^
*f*_star_	Transpiration stream coefficient	-	1e-06–1e-03^a^	
DV	Dispersivity	cm	2–12^f^	
Albedo	Albedo	-	0.05–0.25^a^	
RSMIN crops	Minimum stomatal resistance	s/m	25–100^g^	
RSMIN canola	Minimum stomatal resistance	s/m	25–100^g^	

^a^estimated

^b^Clay-Loam in Gellrich (2014)

^c^Miao et al. (2017)

^d^You et al. (2010)

^e^Milinovic et al. (2015)

^f^Perfect et al. (2002)

^g^Schulze et al. (1994)

Since the parameter space was highly complex and the parameter ranges wide, classical Monte-Carlo-based parameter optimization was not efficient. Thus, we performed two iterations of particle swarm optimization (PSO) as implemented in the hydroPSO package (Zambrano-Bigiarini and Rojas 2013) in GNU R Version 3.4.1. We further employed the R packages Macroutils (Moeys 2015) and HydroGOF (Zambrano-Bigiarini 2017) during the analysis.

In the first PSO iteration, we started 15 chains with 32 particles each and a maximum of 3000 simulations per chain. The simulation results were each evaluated by calculating the Kling-Gupta efficiencies (KGE) (Gupta et al. 2009) of monthly leaching water volumes (*Q*), PFOA concentrations (*C*_PFOA_), PFOA plant uptake (*U*_PFOA_), PFOS concentrations (*C*_PFOS_) and PFOS plant uptake (*U*_PFOS_):

6 $$ {\mathrm{KGE}}_x=1-\sqrt{{\left({r}_x-1\right)}^2+{\left(\alpha -1\right)}^2+{\left(\beta -1\right)}^2} $$

with

7 $$ {\displaystyle \begin{array}{c}\alpha =\frac{s_x(m)}{s_x(o)}\\ {}\beta =\frac{\overline{m_x}}{\overline{o_x}}\end{array}} $$

where *x* includes the target output *Q*, *C*_PFOA_, *U*_PFOA_, *C*_PFOS_ and *U*_PFOS_. *r*_*x*_ is the Pearson correlation coefficient of simulated values *m* and corresponding observed values *o*.${\overline{m}}_x$ and $\overline{o_x}$ are the averages of simulated and observed values and *s*_*x*_(*m*) and *s*_*x*_(*o*) the standard deviations. In order to get a single likelihood value including all *x*, we calculated a multi-target likelihood measure KGE_all_ by averaging all KGE_*x*_.

A threshold value of KGE_all_ > 0.6 was chosen to separate the behavioural from the non-behavioural models after the first iteration of PSO. The minimum and maximum parameter values for all behavioural models were taken as parameter intervals for the second PSO iteration, which was run in 10 chains until convergence or a maximum of 32,000 runs per chain. The relative convergence criterion was taken from the hydroPSO package: the algorithm stops if the absolute difference between the best personal best in the current iteration and the best personal best in the previous iteration was less or equal to the square root of the smallest positive floating-point number of the computer (Zambrano-Bigiarini and Rojas 2013). Finally, all simulations with KGE_all_ > 0.75 were used to calculate a minimum and a maximum uncertainty bound for the output variables *x*.

## Results and discussion

### Simulation results with uncertainty bounds

The 45,000 simulations in the first PSO iteration produced no results with KGE_all_ > 0.60 for scenario I (reversible kinetic adsorption). The best KGE_all_ for scenario I was 0.45. Especially, the concentration simulations were not successful with best KGE values of − 0.54 and 0.36 for PFOA and PFOS, respectively. Thus, scenario I was not optimized further. In the following, the results of scenario II (NER formation) are presented.

The best overall model run of scenario II resulted in a KGE_all_ of 0.80 (median 0.78). The contributions of the specific target variables *x* to KGE_all_ were different among behavioural models: both, PFOA and PFOS concentration calculations, had a median of KGE = 0.89, water percolation KGE = 0.85 and PFOS uptake KGE = 0.84. The uptake of PFOA, however, could not be modelled satisfactorily with a median KGE = 0.48.

#### Leaching

**Fig. 2:**
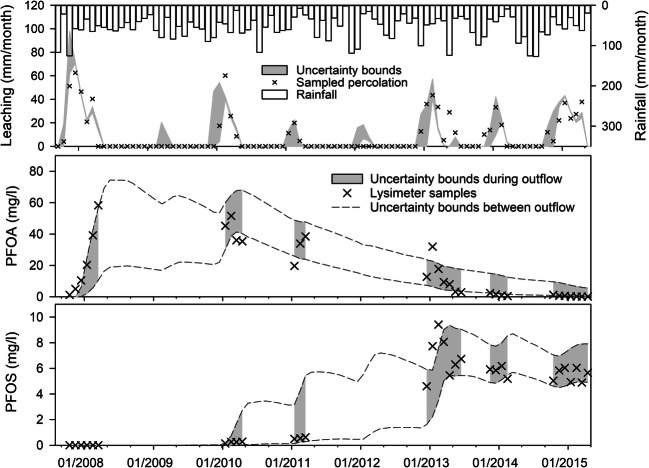
Sampled and simulated monthly percolation (top), PFOA concentrations in leachate (middle) and PFOS concentrations in leachate (bottom) with uncertainty bounds (scenario II). The shaded areas are concentrations during lysimeter outflow; the white areas between uncertainty bounds show the concentrations in the lowest soil layer between outflow events

Generally, monthly percolation is modelled adequately, even though only 75% of the sampled water volumes did fit into the uncertainty bounds (Fig. 2). This might be an effect of the relatively low number of varied parameters affecting percolation: site albedo and stomatal resistance, both influencing evapotranspiration. The result is a relatively narrow uncertainty band. For 2 years, 2009 and 2012, no water percolated down below 1.5 m in the lysimeter, but most behavioural models estimated percolation. This is a hint that either rainfall input was erroneous in these years, or actual evapotranspiration was wrongly calculated in the model. The latter is more likely since evapotranspiration is depending on plant growth in MACRO, which is calculated relatively static with fixed dates between growth states. Plant growth is also not calculated depending on meteorological conditions and nutrient availability. A further issue could be an overemphasis of macropore flow as process bypassing the soil matrix in the MACRO model.

In the model, water in the lowest soil layer builds the vertical outflow of a free-draining lysimeter. Thus, in order to get a picture of PFAAs fate in soil, we calculate the concentrations in the lowest soil layer in addition to the actual percolation events (Fig. 2). For both substances, the dynamics and concentration ranges of the sampled time series could be simulated well. The breakthrough of PFOA was over at the end of the simulation period, while the breakthrough of PFOS was still ongoing. Especially, the PFOS plateau concentrations of the years 2014 and 2015 could be simulated satisfactorily. The width of the concentration uncertainty bounds of both substances was largest during years without lysimeter outflow (2009, 2012). This shows the importance of the information content of samples for the parameter definition and simulation uncertainty in this study. Within sampled years, there is a certain variation in the sampled concentrations, which is best visible at the beginning of 2013 (Fig. 2). These dynamics were not captured by the model and might be a result of high rainfall events of short duration which might have initiated macropore flow in the lysimeter. Even though the MACRO model is capable of simulating macropore flow, it was driven by daily rainfall values in this study. This generally leads to a dampening of short-term rainfall peaks and probably an underestimation of fast macropore flow. Still, most sampled within-year variation was enclosed in the uncertainty bounds.

#### Plant uptake

**Fig. 3:**
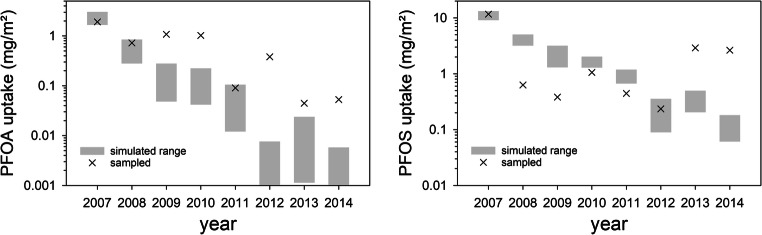
Simulated and observed plant uptake of PFOA and PFOS (scenario II)

Results of the simulated and observed plant uptake (Fig. 3) show that both sampled substance concentrations are mostly outside of uncertainty ranges. The highest uptake of the first year (2007) was covered for both substances. For PFOA, two more years (2008, 2011) and, for PFOS, one more year (2012) were simulated adequately. Simulated plant uptake mainly decreased over time compared with sampled plant uptake, where no definite trend is visible. Still, an increase in plant uptake of PFOS in 2013 and 2014 could be reproduced, but not in the right dimensions. The fact that the highest uptake of the first year could be simulated adequately shows that the model might be used for a worst-case assessment of plant concentrations in the first growing season after a PFAA contamination in the future.

There are several potential reasons for differences between simulated and sampled plant uptake of PFOS and PFOA:

(i). The carry-over of PFAAs to plants depends on factors like specific substance and plant species (Lasee et al. 2019), plant compartment (Stahl et al. 2009) and time that has passed since the substances were applied to the soil (Stahl et al. 2013). Within the model, the transpiration stream coefficient (*f*_star_) is a fixed factor over time, where no crop-related variation is possible. However, due to crop rotation on the lysimeter, time-variable uptake factors would be needed for a more precise simulation.
(ii). All parameters and variables affecting the transpiration stream such as root distribution, root depth and the above-ground plant parameters could have influenced the quality of the result. Even though root depth develops with growth stage in the model, the final root biomass and root depth are the same for each species each year.
(iii). The availability of a substance in the soil water for uptake into roots is also a critical factor. If the vertical distribution of substances in the soil column is not simulated correctly, the plant uptake simulations also suffer from it. Even though we additionally calibrated leaching of substances, substance distribution in different soil layers could not be verified due to a lack of information.

### Substance balance

During the 8 years of simulation, PFAAs masses are distributed between different compartments (solid soil phase, soil water, plants) in the soil-plant continuum (Table 2). The model successfully simulated total leaching mass and total plant uptake (i.e., the uptake sum of all considered years), with the sampled values well within the simulated uncertainty ranges. Considering the substance balance, the PFAA mass transferred into plants was very low (< 0.007%), which suggests that PFAAs leaching is hardly affected by plant uptake. This confirms that phytoremediation is not an effective option for removing PFAAs from agricultural areas (Ross et al. 2018). While the breakthrough of PFOA was over in 2015, 2.4–8.4% of PFOS was still in the mobile adsorption pool and thus potentially leaking out in the following years.

**Table 2:** Simulated and sampled substance balance of PFOA und PFOS from 2007 to 2015 (scenario II)

	mass (g/m^2^)		% of applied	
	PFOA	PFOS	PFOA	PFOS
Leaching mass simulated	4.9–25.5	1.2–3.5	1.4–7.1	0.3–1.0
Leaching mass sampled	13.9	2.7	3.9	0.7
Plant uptake simulated	0.002–0.005	0.016–0.026	0.0006–0.0013	0.004–0.007
Plant uptake sampled	0.005	0.020	0.0013	0.005
Left in Soil (simulated)	334.5–355.1	363.9–366.2	92.9–98.6	99.0–99.7
NER pool	341.8–351.3	335.5–355.3	95.0–97.6	91.3–96.7
Reversible sorption pool	0.0–3.8	8.7–30.7	0.0–1.1	2.4–8.4

In addition to the knowledge gained from the experimental study, the model delivers quantitative information about PFAAs masses in different soil compartments: more than 92% of both substances are in the NER pool after 8 years. This confirms the suggestion of McLachlan et al. (2019) that NER of PFAAs may be built in soil under environmental conditions. However, their reported loss of PFOA (45%) and PFOS (20%) to NER was much lower compared with this study. Still, the timescale of their experiments was much shorter (120 days). Considering that NER formation might be a kinetic process, the much higher NER fraction in this study is reasonable.

### Parameter values

The quantiles of the parameter values of all the behavioural models show that even though we chose a restrictive threshold of KGE_all_ > 0.75, parameter ranges are wide (Table 3). However, clear differences could be found between PFOA and PFOS: The kinetic rate of NER formation was much higher for PFOA (median of 0.0047 1/d) than for PFOS (0.0013 1/d). Also, the 90% intervals show no overlapping for *K*_fOC_ values, which was the case before calibration. There is also a relatively narrow range and a distinct difference of about one order of magnitude for the uptake factor *f*_star_ of both substances.

**Table 3:** Posterior parameter statistics of behavioural models of scenario II (KGE_all_ > 0.75)

	Unit		min	5th	50th	95th	max
*r*_NER_	1/d	PFOA	0.0030	0.0037	0.0047	0.0057	0.0066
	1/d	PFOS	0.0011	0.0012	0.0013	0.0015	0.0016
Freundlich *n*	-	PFOA	0.78	0.78	0.88	1.03	1.13
	-	PFOS	0.65	0.68	0.75	0.89	0.90
*K*_fOC_	ml/g	PFOA	22	30	72	141	156
	ml/g	PFOS	254	256	510	690	893
*f*_star_	-	PFOA	7.7E-06	9.0E-06	1.1E-05	1.4E-05	1.9E-05
	-	PFOS	7.1E-05	8.4E-05	9.6E-05	1.2E-04	1.3E-04
Albedo	-		0.05	0.05	0.11	0.16	0.19
DV	cm		9.6	11.2	12.0	12.0	12.0
RSMIN crops	s/m		34	40	57	79	87
RSMIN canola	s/m		25	25	44	73	100

A classical method for the determination of *f*_star_ is an empirical equation relating *f*_star_ to the octanol-water partition coefficient *K*_OW_, which was originally developed for pesticides (Briggs et al. 1982). However, an experimental determination of *K*_OW_ is not possible for PFAAs due to their water and oil repellent behaviour. Still, a computation by KOWWIN with EPI Suite™ (EPA 2012) and the SMILES code of PFOA and PFOS reveal a log(*K*_OW_) of 4.81 and 4.49, respectively (Fig. 4, supplement). It is noteworthy that a former study suggested KOWWIN unsuitable for highly fluorinated compounds (Arp et al. 2006). Still, KOWWIN has been improved, and the values of the former studies (PFOA, 6.30, and PFOS, 6.28) are not the results of the current version. Using the Briggs equation with the current KOWWIN results, *f*_star_ can be calculated to 0.018 and 0.039, respectively. Due to the high *K*_OW_, these values are lower than for many pesticides (Briggs et al. 1982). Compared with the optimized *f*_star_ values in MACRO, the *K*_OW_-calculated values are three orders of magnitude higher. One reason could be, that, in the lysimeter experiment, the PFAA plant concentrations were only analysed for the above-ground biomass, representing only a fraction of total uptake (Krippner et al. 2014). In a recent study, Schriever and Lamshoeft (2020) explained some of the variability of the Briggs method by different pH values influencing lipophilicity of substances in experimental studies. Still, both, the Briggs method and the model optimization of this study, estimated a higher value and thus a more efficient plant uptake of PFOS compared with PFOA.

According to Table 3, the rate of NER formation is higher for PFOA than for PFOS, which contradicts the current literature on short-term experiments (Chen et al. 2016; Milinovic et al. 2016). However, since more than 90% of both substances is in the NER pool at the end of the simulation period, there is no apparent difference in absolute NER formation of both substances in this study. This is also supported by the fact, that, even though PFOA has a faster NER formation, the availability of the substance for NER formation in the soil is much lower compared with PFOS. This is caused by the calculation concept of NER formation in this study: only adsorbed material can be rendered NER. With a median *K*_fOC_ of 72 ml/g (PFOA) and 510 ml/g (PFOS), the availability of PFOS for NER formation is about 7.1 times higher and the rate of NER formation 3.6 times lower compared with PFOA. Furthermore, a recent lysimeter study supports the fact that NER formation is faster for PFOA than for PFOS in soils under near-natural conditions (McLachlan et al. 2019). A straight-forward method for proving NER existence would be the sampling of the soil profile and the extraction and analysis of the soil for PFAAs residues. However, since the lysimeters contain undisturbed soil columns installed in 1992, a sampling of the soil profile would render these lysimeters useless for other studies. Thus, direct sampling of lysimeter soil is not possible in this case.

### Model structure and applicability

The simulation accuracy of the breakthrough curves and the water percolation of this study is similar to other studies with other organic chemicals (pesticides, veterinary pharmaceuticals) using different leaching models under field conditions (Larsbo et al. 2008; Köhne et al. 2009; Steffens et al. 2013; Kupfersberger et al. 2018), where simulation uncertainties are higher compared with lab studies. Comparing model structures, the dissipation of organic chemicals other than PFAAs was simulated by microbial degradation only. PFAAs, in turn, are not degraded by soil microbes, but NER might be formed in soil under field conditions. However, for veterinary pharmaceuticals, the formation of NER in soil was also proven (Kreuzig and Höltge 2005). Even for pesticides, an irreversible fraction of adsorption is discussed in the literature (Celis and Koskinen 1999; Suddaby et al. 2013). Therefore, the formation of NER is also part of the environmental fate of other organic chemicals in soil, but the process is lumped together with real degradation/transformation into the first-order degradation equation in experimental studies (Kasteel et al. 2010) and leaching models (Larsbo et al. 2008). Thus, since PFAAs are not degradable in soils, the process of NER formation needs to be considered explicitly in leaching models, which was done in this study. An alternative model explaining an apparent irreversibility of the sorption process might be a very slow reversible kinetic sorption site (Suddaby et al. 2013). This process, however, could not explain PFAA behaviour in this study (scenario I).

In contrast to this study, leaching models for organic chemicals were only calibrated against leaching water concentrations and water percolation in the past, even though the models were able to calculate plant uptake of chemicals (Vanclooster et al. 2000; Köhne et al. 2009). Even if plant uptake was simulated, the results were rarely shown (Tiktak et al. 1998). A study simulating plant uptake of organic chemicals during several years after soil contamination could not be found in the literature. One reason might be that for groundwater contamination assessment, plant uptake of organic chemicals is not relevant for all substances, since the uptake mass can be relatively low compared with the leaching masses, which is also the case in this study. For PFAAs, however, even a low plant uptake mass may lead to significant accumulation in human and animal bodies resulting in adverse health effects (Knutsen et al. 2018). Furthermore, this study shows that even low uptake factors after a short-term soil pollution might contaminate cash crops for several years. This may lead to threshold exceedance in field crops, which is currently a problem at agricultural fields in Germany (Brendel et al. 2018).

Unexpected pollution requires fast reaction by authorities in order to guarantee consumers’ safety. In that respect, the use of existing leaching models for emerging contaminants may add to a first and fast environmental assessment strategy. The successful modelling of this study might thus be used in PFOA and PFOS assessment in arable soils of current contamination cases. But, is it possible to use this model also for the other substances in the class of PFAAs? The critical factor would be the simulation of NER formation, which was parameterized by a kinetic rate coefficient in this study. In addition to reversible sorption parameters, the kinetic rate coefficient would have to be derived for each substance from experimental studies, the molecular structure or from soil properties.

## Conclusions

Contamination cases of agricultural fields and other sites by PFAAs were repeatedly reported in the last decade, but tools for the estimation of leaching behaviour of PFAAs in agricultural soils and the carry-over into cash crops are not available. The process of model (and code) development and testing is time consuming and expensive, resulting in a significant time lag between when the model is required (e.g. by authorities) and actual model availability. Therefore, this study explored the use of an existing pesticide-leaching model (MACRO) for simulating PFAAs fate in soil as a possibility for a faster model availability. Since the environmental fate of PFAAs differs from pesticides, a work-around method for NER (non-extractable residues)-formation was required in the model to explain the breakthrough concentrations of PFOA and PFOS in a long-term field lysimeter experiment (scenario II). While other studies implied a correlation between adsorption strength and NER formation, our simulation results suggest a more complex situation: the less adsorptive PFOA was faster transferred to the NER pool than the more adsorptive PFOS. PFOS, however, was better available for NER formation since more substance was adsorbed at equilibrium conditions, and in our model, NER were built from adsorbed substance. Thus, in the field, PFOA might dissipate faster than PFOS, but in the long run (years), the difference might be compensated, and both substances may mainly be present in the NER pool (> 90%). In contrast to the NER scenario, a reversible kinetic rate adsorption model was not applicable in this study (scenario I). Plant uptake could only be successfully described in the first season after soil contamination. Thus, the results of this study led to further research questions that need to be resolved in the future:

(i) Plant uptake modelling largely failed after the first season. Which processes regulate plant uptake of PFAAs and how can these processes be simulated?
(ii) NERs played a crucial role in this study. What are the factors influencing NER formation and kinetics of PFAAs?
(iii) How many PFAAs can be simulated with the same conceptual model of environmental fate processes and how can the parameters be derived?

Despite these open questions, this study showed that it is possible to use existing leaching models for the long-term simulation of the breakthrough curve of PFOA and PFOS in unsaturated soils and the short-term simulation of plant uptake. Even though not all environmental processes are understood yet, these models may already provide valuable information for groundwater protection and crop safety.

## Electronic supplementary material

ESM 1 (PDF 280 kb)

## Data Availability

The model setup and model results are available from the corresponding author on reasonable request.
