# Supplementary material for: Combined leaching and plant uptake simulations of PFOA and PFOS under field conditions
Source: Environ Sci Pollut Res Int. 2020 Aug 31;28(2):2097–107. doi: 10.1007/s11356-020-10594-6 (PMC7785559; doi:10.1007/s11356-020-10594-6)
Supplement: Supplementary file 1 — (PDF 280 kb) [file 11356_2020_10594_MOESM1_ESM.pdf]

Supplementary Material

Combined leaching and plant uptake simulations of PFOS and PFOA under field conditions

M. Gassmann[1], E. Weidemann[1] and T. Stahl[2]

[1]*Department Hydrology and Substance Balance, University of Kassel, Germany*

[2]*Chemical and Veterinary Analytical Institute Münsterland-Emscher-Lippe, Germany*

Contents

4 Tables

1 Figure

Table 4 Plant parameters used in the basic run of the MACRO model

Parameter	Description	Unit	Canola	Winter Wheat	Winter Rye	Winter barley
CFORM[a]	Form factor LAI development	-	2.0	2.0	2.0	2.0
DFORM[a]	Form factor LAI development	-	0.2	0.2	0.2	0.2
BETA[a]	Root adaptability factor	-	0.1	0.1	0.1	0.1
CANCAP[a]	Canopy interception capacity	mm	3	3	3	3
RSMIN[a]	Minimum stomatal resistance	s/m	40	50	50	50
LAIMIN[a]	Leaf are index at ZDATEMIN	-	1	1	1	1
LAIMAX	Leaf are index at IDMAX	-	5[a] / 2.5[c]	6[a]	6[a]	7[d]
LAIHAR	Leaf area index at harvest	-	0.5[b]	2[a]	2[a]	2[a]
ROOTINIT[a]	Root depth at ZDATEMIN	m	0.2	0.2	0.2	0.2
ROOTMAX[e]	Maximum root depth	m	0.9	1.3	1.5	1.2
ZHMIN[a]	Crop height at ZDATEMIN	m	0.2	0.2	0.2	0.2
HMAX[a]	Maximum crop height	m	0.7	0.8	0.8	0.8
IDSTART	Day of crop emergence	Julian day	246	265	265	265
IDMAX	Day of maximum leaf area/root depth	Julian day	156	172	172	172
ZDATEMIN	Day of intermediate crop development	Julian day	60	77	77	77
IHARV	Day of harvest	Julian day	221	233	233	233

[a]FOCUS 2012

[b]Diepenbrock 2000

[c]In 2012 harvested biomass was only half of 2009, thus we assumed also only half the LAI

[d]estimated; barley has a higher LAI than winter wheat (Bach et al. 2016)

[e]estimated in the range reported by Fan et al. (2016)

Table 5 Site Parameters

Parameter	Description	UNIT	Value
ANNTAV	Average annual temperature	°C	9.5
ANNAM	Average annual temperature amplitude	°C	9.3
SNOWMF	Snowmelt factor	mm/°C/d	4
PHI	Site latitude	°	51.3
RINTEN	Average rainfall intensity	mm/h	0.66
ALBEDO	Albedo	-	0.1
ZMET	Height of windspeed measurement	m	15

Table 6 Soil properties as measured in the lysimeter, (Heyn 2013) soil layers were combined (arithmetic average)

Description	Unit	Horizon Ap	Horizon B	Horizon C1	Horizon C2
Depth of soil layer	m	0.1	0.4	0.8	0.2
Clay content	%	17	18.2	26.8	23.8
Silt content	%	79.6	78.9	70.9	73.9
Sand content	%	3.4	2.9	2.3	2.3
pH	-	6.5	6.8	6.9	7.0
Organic carbon content	%	1.5	0.8	0.3	0.2
ρ_s[a]	g/cm³	1.5			

[a]Soil density was assumed based on typical characteristics

Table 7 Soil parameters as calculated by MACRO pedotransfer functions using soil properties from Table 6.

Parameter	Description	Unit	Horizon Ap	Horizon B	Horizon C1	Horizon C2
TPORV	Saturated water content	%	42.16	42.4	42.57	42.6
XMPOR	Boundary water content	%	39.31	40.95	41.57	41.6
WILT	Wilting point	%	13.35	14.66	18.58	16.54
RESID	Residual water content	%	0	0	0	0
GAMMA	Bulk density	g/cm³	1.5	1.5	1.5	1.5
CTEN	Boundary water tension	cm	10	10	10	10
N	van Genuchten's N	-	1.224	1.206	1.157	1.181
KSATMIN	Saturated hydraulic conductivity	mm/h	13.38	3.25	2.59	2.87
KSM	Boundary hydraulic conductivity	mm/h	0.42666	0.40283	0.29776	0.32833
ZN	Pore size distribution factor	-	4	2	6	6
ZM	Tortuosity factor (micropores)	-	0.5	0.5	0.5	0.5
ASCALE	Effective diffusion pathlength	mm	4	4	4	4
ALPHA	van Genuchten's alpha	1/cm	0.008668	0.010155	0.012599	0.011706
TRAP_AIR	Trapped air content	%	0	0	0	0

Fig. 4 Result of calculating K_{OW} for PFOS (1) and PFOA (2) with EPI Suite™

(1)

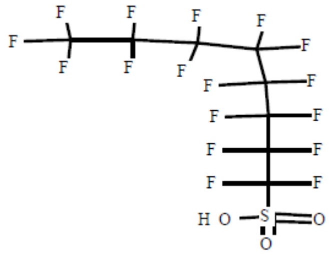

```
Log Kow(version 1.68 estimate): 4.49

SMILES : C(C(C(C(C(F)(F)S(=O)(=O)O)(F)F)(F)F)(F)F)(C(C(C(F)(F)F)(F)F)(F)F)(F)F

CHEM   : Perfluorooctanesulfonic acid
MOL FOR: C8 H1 F17 O3 S1
MOL WT : 500.13
-------+-----+--------------------------------------------+---------+--------
 TYPE  | NUM |        LOGKOW FRAGMENT DESCRIPTION         |  COEFF  |  VALUE
-------+-----+--------------------------------------------+---------+--------
 Frag  |  8  |  C      [aliphatic carbon - No H, not tert] | 0.9723  |  7.7784
 Frag  | 17  |  -F        [fluorine, aliphatic attach]     |-0.0031  | -0.0527
 Frag  |  1  |  -SO2-OH [sulfonic], [coef*(1+0.3*(NUM-1))]|-3.1580  | -3.1580
 Factor|  1  |  SO-C(polyhalo)  structure correction      | 1.4790  |  1.4790
 Factor|  6  |  -CF2(-CF2)(-CF2) (linear -CF2- core)      |-0.2970  | -1.7820
 Const |     |  Equation Constant                         |         |  0.2290
-------+-----+--------------------------------------------+---------+--------
                                                  Log Kow   =    4.4937
```

(2)

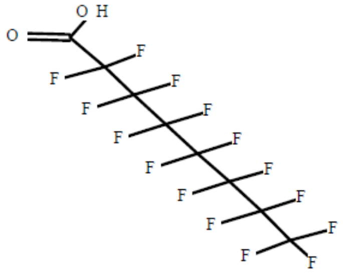

```
Log Kow(version 1.68 estimate): 4.81

SMILES : O=C(C(C(C(C(C(C(C(F)(F)F)(F)F)(F)F)(F)F)(F)F)(F)F)(F)F)O
CHEM   : Perfluorooctanoic acid
MOL FOR: C8 H1 F15 O2
MOL WT : 414.07
-------+-----+--------------------------------------------+---------+--------
 TYPE  | NUM |        LOGKOW FRAGMENT DESCRIPTION         |  COEFF  |  VALUE
-------+-----+--------------------------------------------+---------+--------
 Frag  |  7  |  C      [aliphatic carbon - No H, not tert] | 0.9723  |  6.8061
 Frag  | 15  |  -F        [fluorine, aliphatic attach]     |-0.0031  | -0.0465
 Frag  |  1  |  -COOH     [acid, aliphatic attach]         |-0.6895  | -0.6895
 Factor|  5  |  -CF2(-CF2)(-CF2) (linear -CF2- core)      |-0.2970  | -1.4850
 Const |     |  Equation Constant                         |         |  0.2290
-------+-----+--------------------------------------------+---------+--------
                                                  Log Kow   =    4.8141
```

Literature

Bach H, Migdall S, Brüggemann L, Brohmeyer F, Bönewitz U, Buddeberg M (2016) Satellitengestützte Ertragserhebung. Publication series of the State Office for Environment, Agriculture and Geology Saxony 2016(21)

Diepenbrock W (2000) Yield analysis of winter oilseed rape (Brassica napus L.): a review. Field Crops Research 67(1):35–49. doi: 10.1016/S0378-4290(00)00082-4

Fan, J., McConkey, B., Wang, H., Janzen, H., 2016. Root distribution by depth for temperate agricultural crops. Field Crops Research 189 (Supplement C), 68–74.

FOCUS (2012) Generic guidance for FOCUS surface water scenarios

Heyn J (2013) Comparison of Management Models - Lysimeter Experiment in Kassel-Harleshausen. Aspects: Productivity, water and nitrogen efficiency. In: Wirkung unterschiedlicher N-Düngungsstrategien auf Ertrag und Qualität bei Weizen und Raps–langjährige Ergebnisse aus der Landesforschung in Sachsen, pp 44–66
